# Trace Element Concentrations in Tree Leaves and Lichen Collected Along a Metal Pollution Gradient Near Olkusz (Southern Poland)

**DOI:** 10.1007/s00128-017-2219-y

**Published:** 2017-11-27

**Authors:** Marta Zakrzewska, Beata Klimek

**Affiliations:** Faculty of Biology and Earth Sciences, Institute of Environmental Sciences, Jagiellonian University, Gronostajowa 7, 30-387 Kraków, Poland

**Keywords:** Air pollution, Atomic absorption spectrometry (ASA), Bioindicator, Environmental monitoring, *Hypogymnia physodes*

## Abstract

The aim of the study was to assess the metal pollution in the vicinity of the Bukowno smelter near Olkusz in southern Poland. Birch and oak leaves, pine needles and a lichen *Hypogymnia physodes*, overgrowing pine bark were collected at stands at different distances from the smelter and analysed for cadmium (Cd), copper (Cu), lead (Pb) and zinc (Zn) content. Concentrations of metals in the lichen were usually higher than in the tree leaves/needles and decreased with distance from the smelter, apart from the Cu content. The strongest correlation was noticed between Cd and Pb concentrations, which indicates a common pollution source (the smelter). Our results show that birch leaves can be potentially useful as a bioindicator of Zn air pollution since this species was shown to accumulate high amounts of zinc, related to environmental pollution with that metal, in their leaves.

Industrial heavy metal pollution has become a serious environmental problem all over the world. Monitoring of metal pollution levels in the air, soil and water is commonly complemented with biological monitoring, consisting of the measuring of metal content in living organisms. One of the most widespread monitoring organisms are lichens – slow-growing associations of fungi and green algae or cyanobacteria (Latkowska et al. 2005). Lichens are known to accumulate some airborne pollutants by both wet and dry deposition (Nash 1996; Asplund et al. 2015). Lichens are excellent model organisms for the environmental monitoring of air metal pollution on account of their relative resistance to metal pollution and their ability to accumulate metals both from the ground and directly from the air (Sawicka-Kapusta et al. 2010; Kularatne and de Freitas 2013; Will-Wolf et al. 2017). Accumulated trace metals do not interfere noticeably with the lichens’ cellular processes, so these organisms are widely used in mapping spatial and temporal patterns of trace metal fallout (Klimek et al. 2015; Węgrzyn et al. 2016). However, lichens are sensitive to sulphur dioxide causing the most visible consequences, referred to as *lichen deserts*, which occur in cities and industrial regions throughout much of the world (Lisowska 2011). Such a state may make the use of lichens as bioindicators difficult or even impossible in some of the most polluted areas, e.g. those located close to industrial plants. In fact, the majority of regions of high industrialisation and urbanisation are polluted with multiple substances, often including both metals and sulphur oxides.

Upper Silesia in southern Poland is one of the most contaminated areas of Europe and is often referred to as an area of ecological disaster (Pawlowski 1990). Metal ore deposits and intensive mining and smelting have resulted in high metal contents in environment components, achieving values as high as 5437 mg kg^−1^ soil dry weight (DW) of total zinc concentration, 1877 mg kg^−1^ soil DW of lead and 57 mg kg^−1^ soil DW of cadmium (Klimek 2012; Klimek et al. 2016). These values exceed the values accepted by a Polish regulation as non-harmful several times over.

Lichens are known to be excellent indicators of air pollution whereas plants, including trees, absorb metals from both the air and soil through foliar and root uptake (Kozlov et al. 1995; Shahid et al. 2017). Therefore, the metal contents in lichens and tree leaves/needles collected in these same places may differ significantly. As such, the authors assume that common plant species can be used for environmental monitoring in such areas as long as the metal concentration in the measured organisms will be related to environment pollution, which is the most essential feature of any model bioindicator.

The aim of this study was to assess current trace metal pollution in the Olkusz region using the lichen *Hypogymnia physodes*, a widely used bioindicator, as well as selected tree species that are common to the region – silver birch *Betula pendula*, sessile oak *Quercus robur* and Scots pine *Pinus sylvestris*. We were searching for the tree species that follow the pattern of metal concentration in lichen, and therefore would be potentially useful as bioindicators of air pollution in heavily polluted areas.

## Materials and Methods

The samples of lichen, leaves of birch and oak and pine needles were collected along a metal pollution gradient near Olkusz in southern Poland in the vicinity of the former zinc and lead smelter. Mining and smelting activity have been recorded here since the Middle Ages and large-scale industry in the Olkusz region started in the 1970s. The samples were collected on forest stands located 2.5–12 km from the Bukowno smelter, that is between 50°16′ and 50°45′N and 19°37′–18°39′E. The pollution level in the region is well known based on several earlier studies (Laskowski and Maryański 1993; Niklińska et al. 2006; Hrdlička and Kula 2011; Klimek et al. 2016). The relatively short gradient was established (up to 12 km from the smelter) towards the west (the main wind direction and air pollutants transportation). The samples were collected during 1 day in December 2014.

Each lichen sample was composed of lichen collected from the bark of a neighbouring few pine trees (*P. sylvestris* L.) using a knife and mixed in a single bag. All the samples were collected from pine as tree species differ in elemental composition, which affects the element composition in lichen thalli (Asplund et al. 2015). The leaves of silver birch *B. pendula* Roth. and sessile oak *Q. robur* as well as the needles of Scots pine *P. sylvestris* L. were collected from the west side of a few neighbouring trees. In total, 72 samples of lichen and 108 samples of tree leaves or needles were collected.

The samples were transported to the laboratory in bags. Then, the samples were purged to remove any bark or dust particles, crumbled and dried at 60°C for 5 days to achieve a constant mass (dry weight, DW). The total concentrations of the metals (Cd, Cu, Pb, Zn) in each sample were determined after wet digestion of 0.5 g of DW of sample in 10 mL of SupraPure-concentrated HNO_3_ and HClO_4_ (4:1 v/v) (Sigma-Aldrich) (Klimek et al. 2015). The metal concentrations in the digests were measured using atomic absorption spectrometry (AAS) with a flame or graphite furnace nebuliser (Perkin-Elmer). To control for efficiency of the digestion, purity of the chemicals and glassware, and precision of the analytical equipment, five blank samples and five replicates of standard certified reference material were processed along with the lichen samples (Trace elements in lichen, CRM^®^-482; IRMM, Geel, Belgium) (Klimek et al. 2015). Each analysis was performed in two replicates and the data were averaged and expressed based on the DW of the sample. The detection limits were determined according Perkin-Elmer guidelines, that is after multiple metal analysis of deionised water being zero concentration of calibration curve and then calculation for the mean value with its triple standard deviation.

The normality criterion for data distribution within groups was checked with the Shapiro–Wilk test and the data were transformed when necessary. The General Linear Model was used to test for the differences in mean values of metal concentrations in the lichen and tree leaves/needles (categorical factor), and to test for the effect of distance from the smelter (quantitative factor) and for interaction between the two (*p* < 0.05). In cases where the interaction was not significant, it was removed from the model. A Comparison of Regression Lines was used to find differences in the slopes between the studied sample type (lichen and tree species) (*p* < 0.05). Correlation analysis was used to test the relationships between the concentrations of the studied metals, and Pearson’s correlation coefficients were considered significant at *p* < 0.05. Clustering method, a method based on nearest neighbour with squared Euclidean distance, was used to detect similarity pattern in metal concentrations in the lichen and leaves/needles that could possibly indicate a common source of pollution. All statistical analyses were performed using the Statgraphics Centurion XVII (StatPoint Technologies Inc., Warrenton, VA, USA).

## Results and Discussion

The detection limits for the analytical methods used were 0.02, 0.04, 0.36 and 8.75 mg kg^−1^ for Cd, Cu, Pb and Zn, respectively. Metal recoveries were 99%, 94%, 93% and 99% for Cd, Cu, Pb and Zn, respectively. For each metal the GLM model was highly significant (*p* < 0.0001) (Table 1). The lichen and tree leaves/needles differed in each metal content, whereas the metal content in the collected material decreased with distance from the smelter for Cd, Pb and Zn, but the distance effect was not significant for Cu (Table 1). The only significant interaction between species and distance from the smelter was found for Pb (Table 1), which suggests strong differences between the species in Pb accumulation along the pollution gradient.

**Table 1:** GLM parameters for metal concentrations in lichen thalli and tree leaves/needles collected along the pollution gradient near the smelter

	Metal			
	Cd	Cu	Pb	Zn
Model; *p* value	< 0.0001	< 0.0001	< 0.0001	< 0.0001
Model; ${\text{R}}_{{{\text{adj}}}}^{{\text{2}}}$ (%)	72.6	46.6	83.2	55.4
Factor: species (lichen or trees); *p* value	< 0.0001	< 0.0001	< 0.0001	< 0.0001
Factor: distance from smelter; *p* value	0.0027	0.2441	0.0010	0.0001
Interaction species x distance; *p* value	ns	ns	0.0088	ns

Differences in mean metal concentrations between species were found for each element despite the relatively high variance in the data; significantly higher Cd and Pb concentrations were found in the lichen thalli compared to the tree leaves/needles (Table 2). The concentrations of Zn were similarly high in the lichen thalli and birch leaves compared to the other two species (Table 2). In turn, the highest concentrations of Cu were found in the oak leaves (Table 2).

**Table 2:** Trace metal concentrations (mg kg^−1^ DW) in lichen thalli and tree leaves/needles collected in the studied sites; mean values (± standard deviation; n = 18 for the lichen or n = 9 for the leaves/needles)

Species	Metal concentration (mg kg^−1^ DW)			
	Cd	Cu	Pb	Zn
Lichen	3.16^**c**^ (± 2.40)	8.47^**b**^ (± 2.07)	51.47^**b**^ (± 62.76)	470.8^**b**^ (± 286.3)
Birch	0.56^**b**^ (± 0.30)	5.89^**ab**^ (± 1.67)	2.48^**a**^ (± 2.11)	761.1^**b**^ (± 532.9)
Oak	0.17^**a**^ (± 0.14)	14.4^**c**^ (± 8.06)	2.15^**a**^ (± 0.71)	196.2^**a**^ (± 243.8)
Pine	0.64^**b**^ (± 0.74)	4.82^**a**^ (± 2.62)	2.21^**a**^ (± 1.18)	229.2^**a**^ (± 205.4)

Results of GLM analysis: differences between materials (columns) are indicated with different bold letters in superscript (a–c)

**Fig. 1:**
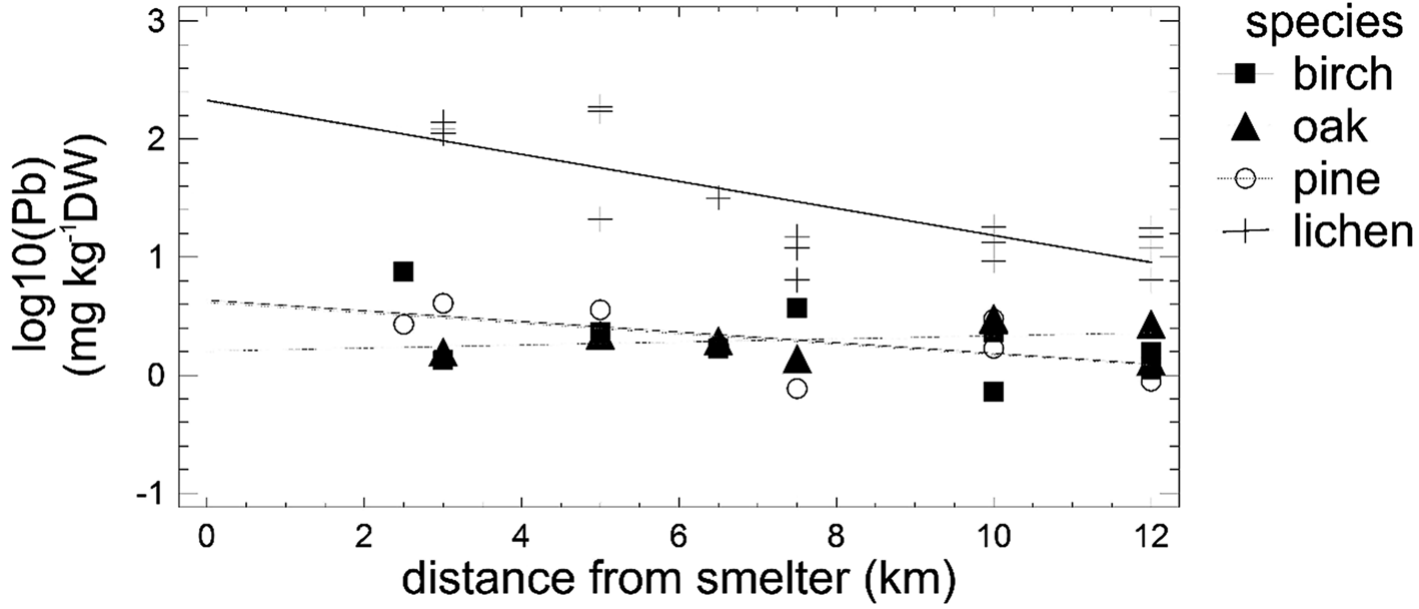
A comparison of regression lines for the Pb content in the lichen and tree leaves/needles and its relation to the distance from the smelter (intercepts *p* < 0.0001, slopes *p* = 0.0088)

The Comparison of Regression lines analysis indicated that Pb concentration decreased with distance from the smelter especially in the lichen, whereas it did change greatly in the tree leaves/needles along the metal pollution gradient (Fig. 1). For the other metals, the decrease in their concentration with distance from the smelter was similar for all the studied species (data not shown).

**Fig. 2:**
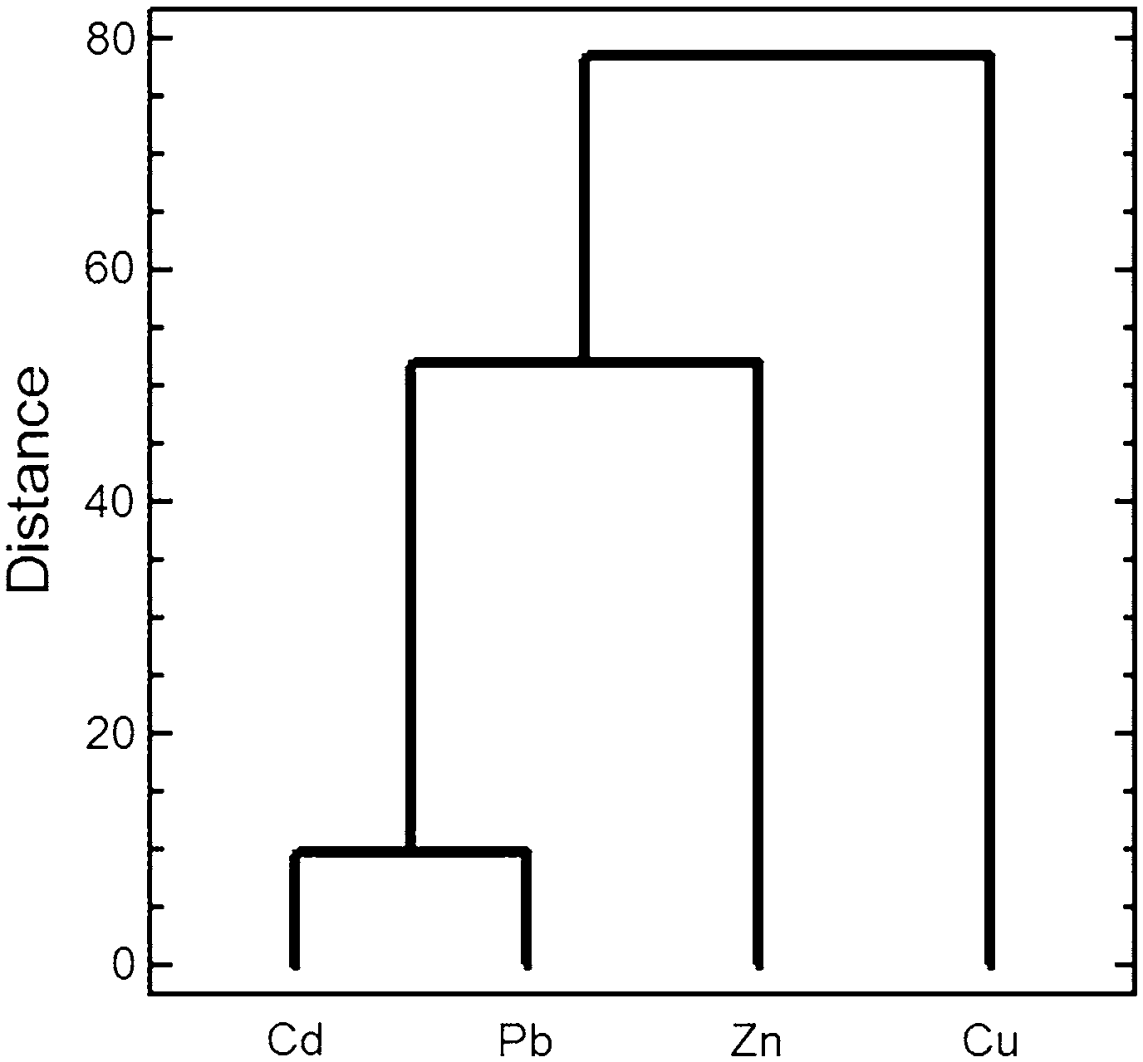
Dendrogram presenting the similarity between metal concentrations (Cd, Cu, Pb, Zn) in the lichen and studied tree leaves/needles

The Cd and Pb exhibited a similar pattern in their concentrations in the collected samples as shown by the cluster analysis (Fig. 2). These included the highest metal content in the lichen and a much lower content in the tree leaves/needles, especially in the oak leaves (Table 2). The Zn concentration pattern was slightly different from Cd and Pb, as the highest metal content was found both in the lichen and birch (Fig. 1; Table 2). In turn, the Cu concentration pattern differed from all the others (Fig. 2), which resulted from the lower Cu content in the lichen than in the oak leaves and a lack of effect of distance from the smelter (Tables 1, 2). All this indicates that Cu sources in the collected material were other than from the smelter. In fact, metal ores in the Upper Silesia Industrial region contain zinc, lead and cadmium but not copper and, moreover, copper ores are not processed in the region.

Our results indicate that birch leaves can be potentially useful as a bioindicator of Zn pollution since this species was shown to accumulate high amounts of Zn in their leaves, which is related to environmental pollution from that metal. In our study we found Zn concentration in the birch leaves reached as high a value as 1866 mg kg^−1^ DW in the closest vicinity of the metal smelter. This result is similar to the 1640 mg kg^−1^ DW obtained there previously by Hrdlička and Kula (2011). Birch resistance to pollution makes it a possible bioindicator in environmental studies because it survives even within wastelands (Kozlov et al. 1995). Soil pH can influence the solubility of metals, and metal contamination typically reduces soil pH (Speir et al. 1999; Boivin et al. 2006). Birch has been found to raise soil pH (Priha and Smolander 1999) and this may support soil microorganisms that protect the tree against metals contamination. Compared to other tree species, birch tends to accumulate Zn even in regions unpolluted or moderately polluted with metals (Kosiorek et al. 2016). Since Zn is a microelement this means that in some doses it is beneficial for organisms, including plants (Kozlov et al. 1995; Shahid et al. 2017).

In addition, we found that birch and pine can be used potentially as bioindicators for Cd. However, Cd concentrations in birch leaves and pine needles are much lower than those in lichens. This may generate problems with low correctness of metal analysis and small differences between polluted and unpolluted locations. In the case of Pb, we showed definitively that only lichens can serve as air quality indicators of Pb pollution.
